# Design and Test of a New Inductive Force Sensor

**DOI:** 10.3390/s18072079

**Published:** 2018-06-28

**Authors:** Robert Bram Giesberts, Victor IJzebrand Sluiter, Gijsbertus Jacob Verkerke

**Affiliations:** 1 Department of Biomechanical Engineering, University of Twente, Drienerlolaan 5, 7522 NB Enschede, The Netherlands; r.b.giesberts@utwente.nl (R.B.G.); g.j.verkerke@gmail.com (G.J.V.); 2 Department of Rehabilitation Medicine, University of Groningen, University Medical Center Groningen, Hanzeplein 1, 9713 GZ Groningen, The Netherlands

**Keywords:** force sensor, induction, LDC, drift

## Abstract

The currently accepted interval of weekly cast changes in the treatment of clubfeet seems unsubstantiated. A force sensor is needed to determine the adaptation rate of a clubfoot to establish what cast change interval would be most effective and efficient. We developed a force sensor based on the principle that the resonance frequency of an LC-tank changes when a metal target is brought in close proximity. A thin rubber ring between the LC-tank and the metal target transformed this proximity sensor into a force sensor. With a static load test and an incremental load test, the performance of the constructed force sensors was characterized. The custom-made sensor showed excellent sensitivity (($1.7\pm 0.8\times 10^{5}$) counts/N), resolution ((0.15±0.06) mN), and accuracy ((3.5±3.0) %) for the application. The observed drift was (2.1±0.7) %/log${}_{10}$(h), which is lower than other thin force sensors. Preliminary results of measurements in the treatment of Dupuytren fingers and clubfeet show good functioning for long-term force measurements.

## 1. Introduction

**Figure 1:**
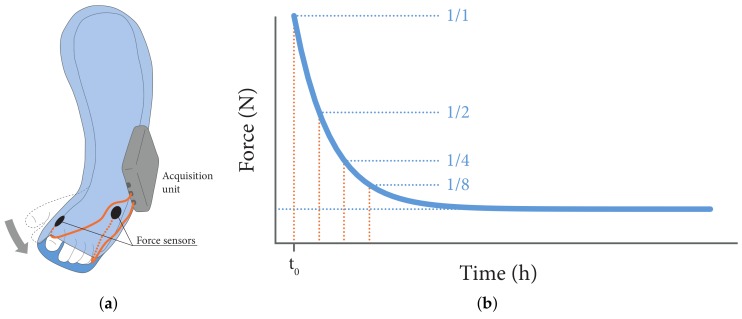
(**a**) Sensor location: The clubfoot is fixated in a corrected position with a plaster cast. (**b**) Expected plot: Initially the foot presses against the cast but over time this force decreases due to adaptation of the biological tissues.

A clubfoot is a congenital deformity of the foot which is commonly treated with the Ponseti method [1]. In this treatment, in the first weeks after birth, the foot is manipulated towards a more corrected position and fixated with a plaster cast (Figure 1a). This weekly procedure is repeated several times until the deformity is fully corrected. At first the foot resists the newly imposed position by pressing against the rigid plaster cast, but over time the biological tissues inside the clubfoot adapt to the new position: the force against the plaster cast decreases (Figure 1b). The adaptation rate of a clubfoot can be determined by measuring the decrease of force between the cast and the clubfoot. This information is essential to improve the treatment method because it can determine when the next correction is due. This could be much faster than after one week.

To measure the adaptation rate of the clubfoot, a force sensor is needed with very specific requirements. The specific application that is described above requires a sensor that is thin enough to fit underneath the cast without causing any damage to the skin, accurate enough for a measurement over the period of one week and its power should be supplied by a small battery that would be safe in a portable device for a baby. The requirements used for the development of the force sensor described in this paper are summarized in Table 1.

Numerous force sensors with varying functional principles are commercially available, but, to the best of our knowledge, none of them are suitable for long-term precision measurements within a portable setting. Other sensors do not fit our requirements because they are either too bulky or experience too much drift. OEM load cells (e.g., Futek Inc., Irvine, CA, USA) are too large for our requirements, and it would be challenging to make a low-power amplifier with high resolution for load cells based on strain gauges. Force Sensitive Resistors (FSR, e.g., FlexiForce, Tekscan, Boston, MA, USA) are susceptible to drift [2,3,4]. OptoForce sensors (OptoForce, Budapest, Hungary) are elegant yet not thin enough to be placed below the plaster cast. TakkTile sensors (RightHand Robotics, Somerville, MA, USA) are a relatively good candidate, but we found that their measurement range is difficult to adjust and their accuracy over long-term measurements is unknown.

The aim of this paper is to describe the performance and accuracy of a new inductive force sensor for the application of long-term force measurements in a portable setting.

## 2. Sensor Design

An inductive force sensor was developed based on the evaluation kit of the inductance to digital converter LDC1000 (Texas Instruments, Dallas, TX, USA) and its successor LDC1614 [5]. This integrated circuit is able to accurately determine the resonance frequency ($f_{sensor}$) of an LC-tank. Its main output parameter is LHR_DATA, a digital value that is proportional to frequency, with 28 bits of measurement resolution. The resonance frequency is influenced by the distance of the coil to any metal target. In most applications, a non-ferromagnetic and conductive metal (e.g., aluminum) is used to modulate the inductance of the coil. Closer proximity of the target to the coil will increase eddy currents in the target, thereby decreasing the inductance of the coil. In close proximity, the relation between distance and resonating frequency can be linearized with small errors. The exact functioning and examples for sensor design can be found in the documentation provided by Texas Instruments e.g., [6,7].

To convert this proximity sensor into a force sensor, an elastic medium with a known stiffness was added between the coil and the target. Our initial approach consisted of a simple sandwich of these three components. Although this solution has been researched by others [8], and patented by others a.o., [9,10], it did not appear as a commercial product at the time of writing of this manuscript. Preliminary tests with this approach showed a high accuracy and sensitivity but mediocre long-term results, with time drift values as high as 50%/day. Several tests were conducted to identify the cause for this drift. If the sensor was not loaded with any weight, no drift was observed. Creep of the rubber medium was rejected with a creep test under 50% compression for 14 days, which showed no deformation. Replacing the compressible medium by an equally thin sheet of (incompressible) glass did not resolve the drift. Neither did replacing the aluminum target for a polyethylene target. One of the few hypotheses remaining was plastic deformation of the PCB.

**Figure 2:**
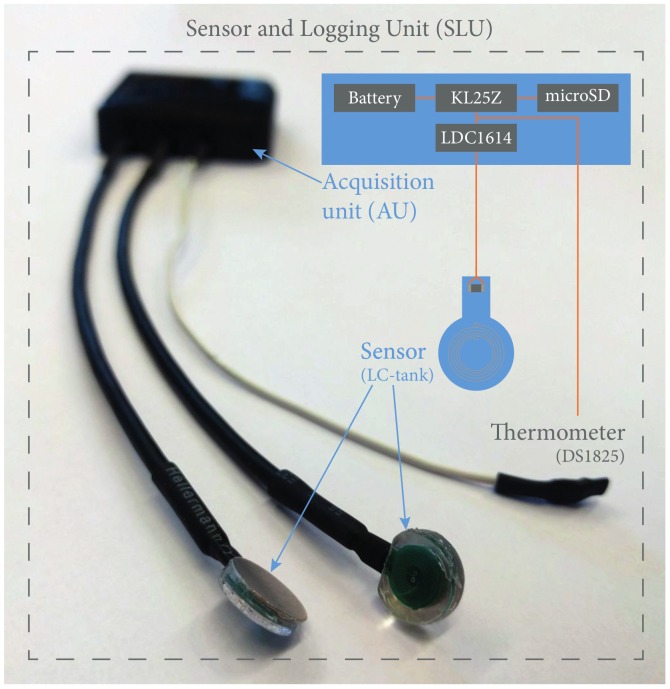
Two sensors and an acquisition unit (AU). The AU (top) allows the connection of two sensors (below) and a thermometer (right). Schematic representation of a sensor and logger to explain the distinction between sensor, AU, and the combination of the two: the sensing and logging unit (SLU).

**Figure 3:**
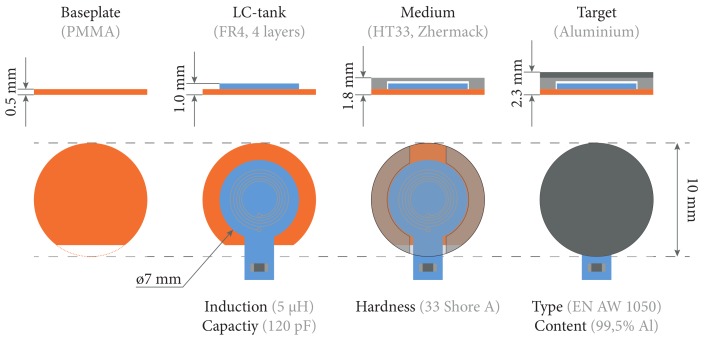
Sensor specifications. Front and top view of the sensor in four stages of the assembly process. The sensor is built out of four parts of which the technical specifications are given here.

In the final design, a base-plate was added and the rubber medium was shaped into a “ring”, enclosing the PCB (see Figure 2 and Figure 3). With this approach, loading the sensor compresses the rubber ring around the PCB but prevents loading—and possibly deforming—the PCB. With the rubber ring, this solution is a compromise on the size (ø10 vs. ø7 mm). A DS1825 thermometer [11] was added to correct for the influence of temperature.

The terminology used for the sensor, the acquisition unit (AU), and their combination as a sensing and logging unit (SLU) throughout this paper are presented in Figure 2.

### Acquisition Unit

The sensor was connected to a custom-made AU to log LHR_DATA over time. The AU was equipped with the LDC1614 [5] and a KL25Z microprocessor [12] that was programmed to write the collected data to a microSD memory card. The AU was powered with a small single cell Lipo battery (165 mAh).

Programming of the AU was optimized for high sensitivity, low power consumption, and little time drift. This means that for the internal settings of the LDC1614, CHx_RCOUNT was set to maximum (0xFFFF) for increased precision at the expense of bandwidth, SETTLECOUNT_CHx to 50 (to allow a sensor settling time of $t_{settle}$ = 20 ms), and CHx_DRIVE was automatically determined using the method described by Holubeva [13] and set to 23. The resulting sample rate was 18 Hz. The used code is available online [14].

## 3. Experimental Section

### 3.1. Data Collection

To reduce energy consumption, the AU was programmed to write packages of 60 data samples at a time to the microSD card, rather than sample-per-sample. The thermometer was programmed to do one temperature measurement per package.

### 3.2. Test Setup

**Figure 4:**
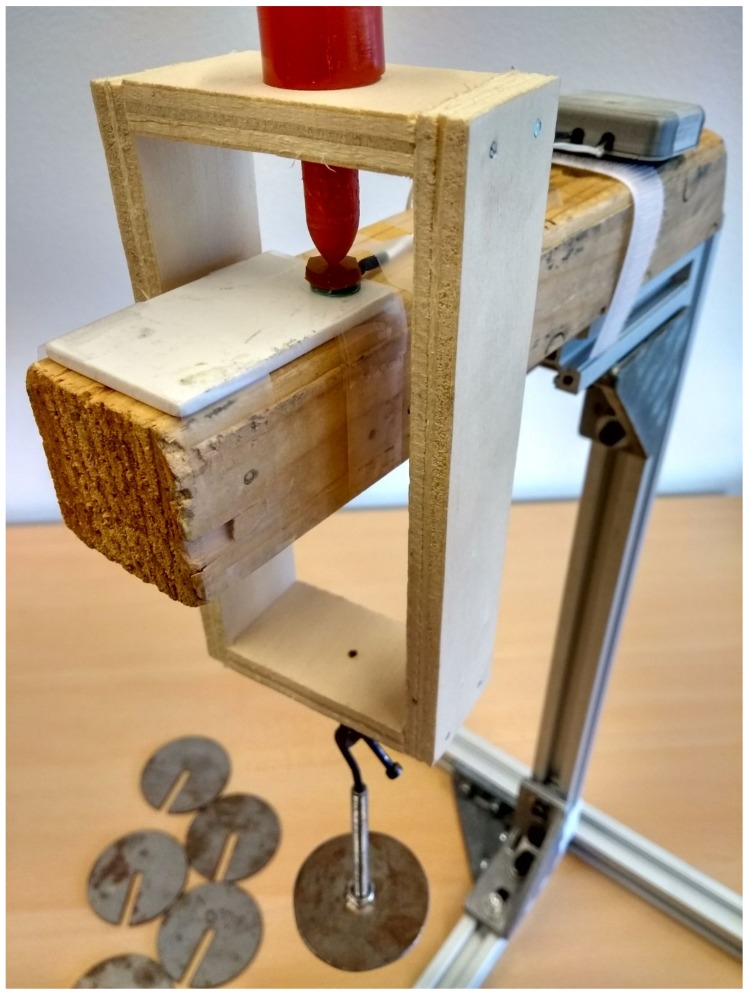
Calibration rig. Each sensor was placed in the calibration rig. Steel disks of 100 g each (bottom left) were placed on the hanger to apply a load.

A calibration rig was fabricated which allowed us to hang known weights on the sensor. The applied load (*F*, in N) compresses the sensor, thereby changing the oscillation frequency. LHR_DATA, proportional to this frequency, is measured and stored by the AU. None of the parts used in close proximity of the sensor were made of metal to prevent any interference with the LC-tank. A hanger of 127 g was used as a pre-load to which weights of 100 g each were attached (see Figure 4).

### 3.3. The Static Load Test

A static load test was performed to carefully assess time drift of the SLU. The SLU was placed in the calibration rig with the pre-load in place. During the first 2 h, the force sensor was not loaded, then a weight of 500 g was hung on the force sensor and left there for at least 12 h after which the load was removed. Measurements continued for another 2 h.

The following characteristics were determined:

*Noise* was defined as the standard deviation of the output signal (LHR_DATA), filtered with a high-pass filter (0.01 Hz). Furthermore, the noise performance was characterized by measuring the Allan deviation [15] (p. 15) as a function of averaging time using a sensor that was not loaded for eight hours.

*Temperature drift* of the SLU was defined as the change in LHR_DATA per change of the room temperature (heating of the empty office used for our tests was controlled centrally and caused fluctuations in temperature). It was calculated as ΔLHR_DATA/ΔT over the period while the sensor was loaded and the input parameter (*F*) was constant, in counts/${}^{\circ }$C.

*Time drift* of the SLU was defined as the relative change of LHR_DATA over time, after a 30 s settling time. Two calculation methods were used:

- Drift was calculated as the gain of the best linear fit of the signal over logarithmic time, in %/log(h) [16].
- Drift was calculated as the relative difference between the initial value and the final value after several specific times [3].

### 3.4. The Incremental Load Test

The SLU was loaded with incremental 30-second-long steps of 100 g each up to a maximum of 1 kg. With this test, the following characteristics were determined:

*Sensitivity* of the SLU was defined as the change of the output signal per change of input parameter (ΔLHR_DATA/ΔF) [17]. It was determined as the gain of a best linear fit in counts/N. *Non-linearity* was defined as the greatest deviation to this linear fit [17].

*Accuracy* of the SLU was defined as the greatest deviation to a polynomial fit that was used to allow conversion of LHR_DATA to force, as percentage of the full scale output.

*Hysteresis* of the SLU was defined as the maximum difference between the loading and unloading curves, as percentage of the full scale output.

### 3.5. Data Processing

The collected data were processed using Matlab R2017b. In post-processing, the LHR_DATA for the static load tests were corrected for an apparent start-up effect and for the influence of temperature changes using the identified temperature drift.

#### Start-Up Effect

**Figure 5:**
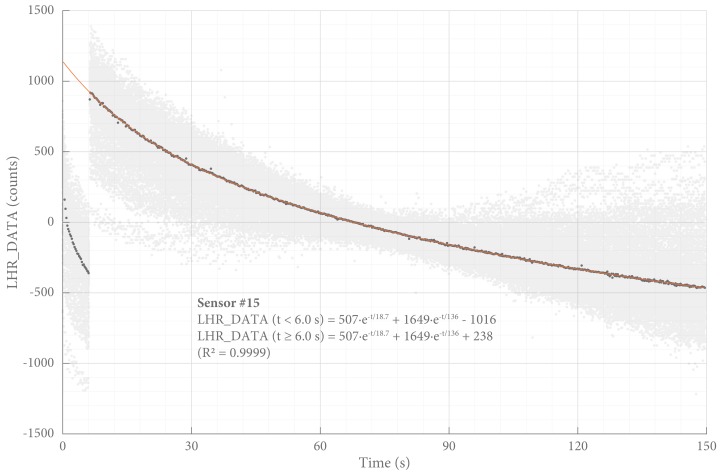
Start-up effect. The output signal gradually decreases every time the SLU is started. For this graph, the SLU was programmed to take ten 150 s measurements per hour, (gray). For all timestamps, the mean was calculated (dark gray), and least squares regression was used to find the best fit to a double exponential function (orange). The typical sensitivity (Table 2) can be used to convert LHR_DATA to a measure for force: 1500 counts approximate 0.01 N.

In its intended application in force measurements in the treatment of clubfoot, the SLU uses a logging protocol of four 30 s periods per hour to preserve battery power. Every time the SLU is activated an initial start-up effect is observed, as shown in Figure 5. Seemingly independent of external influences, the output signal consistently decreases over time to reach a plateau after several minutes. This behavior was observed in all SLUs with almost identical parameters. For all sensors, this behavior was identified, quantified, and corrected.

## 4. Results

**Figure 6:**
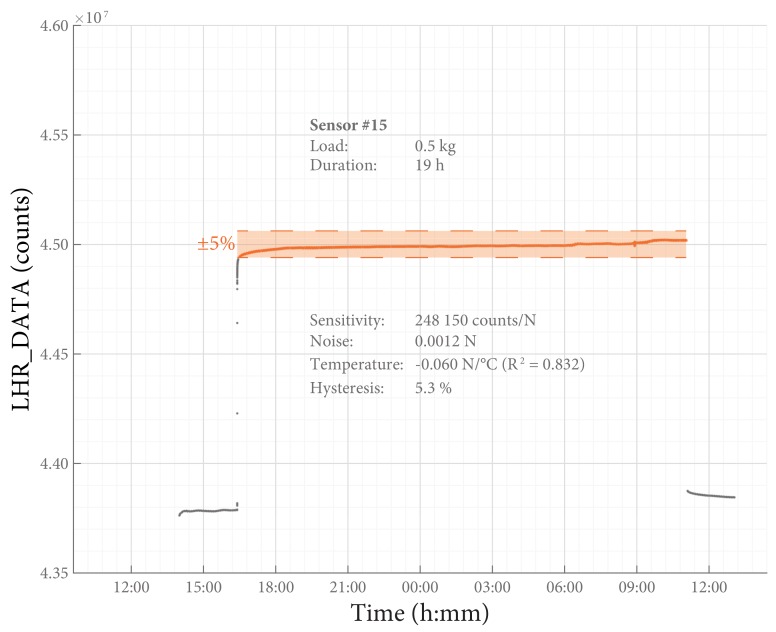
Static load test. The results of a typical static load test. The typical sensitivity (Table 2) can be used to convert LHR_DATA to a measure for force: 1500 counts approximate 0.01 N.

**Figure 7:**
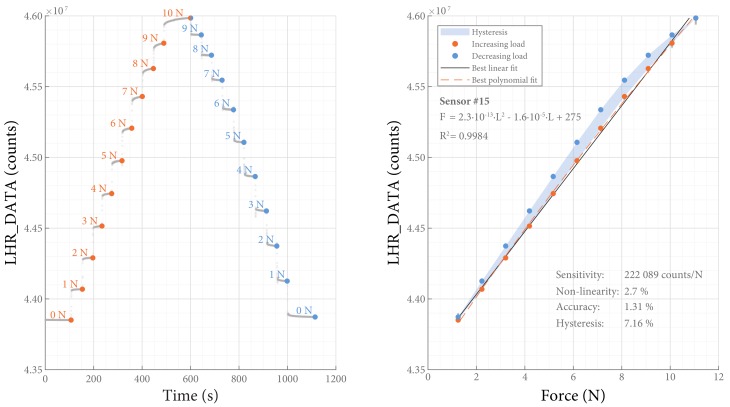
Incremental load test. The results of a typical incremental load test.

The static load test was performed with 9 sensors and the incremental load test with 13. Typical examples of the resulting data of both tests are presented in Figure 6 and Figure 7, respectively. The performance of the inductive force sensors are summarized in Table 2 and described in more detail below.

**Figure 8:**
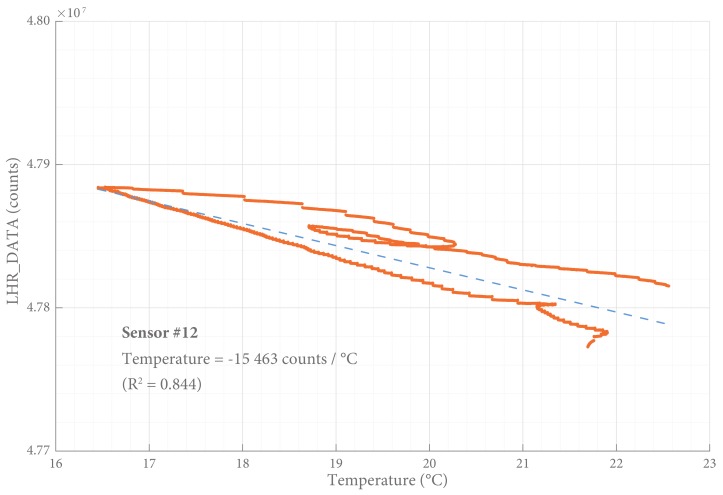
Temperature influence. About 85 % of the changes in output signal can be explained by fluctuations in temperature. It is not clear if this temperature influence is independent of the observed time drift and the load applied. The typical sensitivity (Table 2) can be used to convert LHR_DATA to a measure for force: 1500 counts approximate 0.01 N.

The influence of temperature was (−0.088 ± 0.030) N/°C ($R^{2}$ = 0.84). The results of a typical temperature analysis are shown in Figure 8.

**Figure 9:**
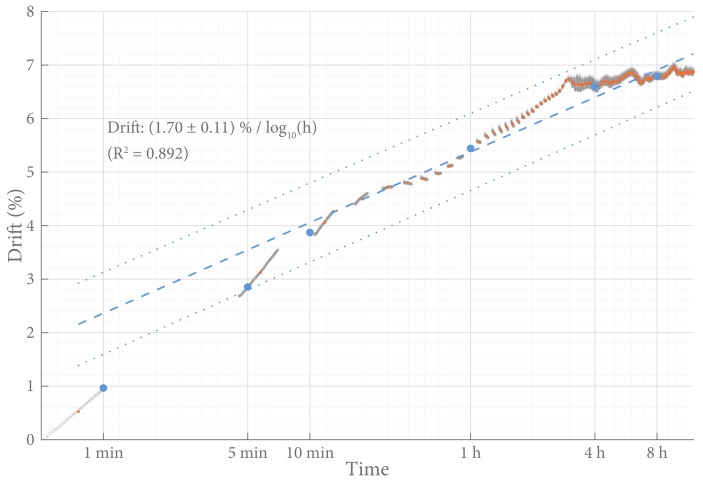
Time drift. The results of a typical static load test, during the loaded period, with time on the *x*-axis on a logarithmic scale. Orange dots represent the observed drift. A best linear fit and its 95% prediction interval is presented as a blue line. The blue dots represent the drift at specific times, as per Parmar et al.’s [3] method.

The time drift of the SLU was determined to be (2.1 ± 0.7) %/log${}_{10}$(h). Figure 9 shows the observed drift during a typical incremental load measurement.

**Figure 10:**
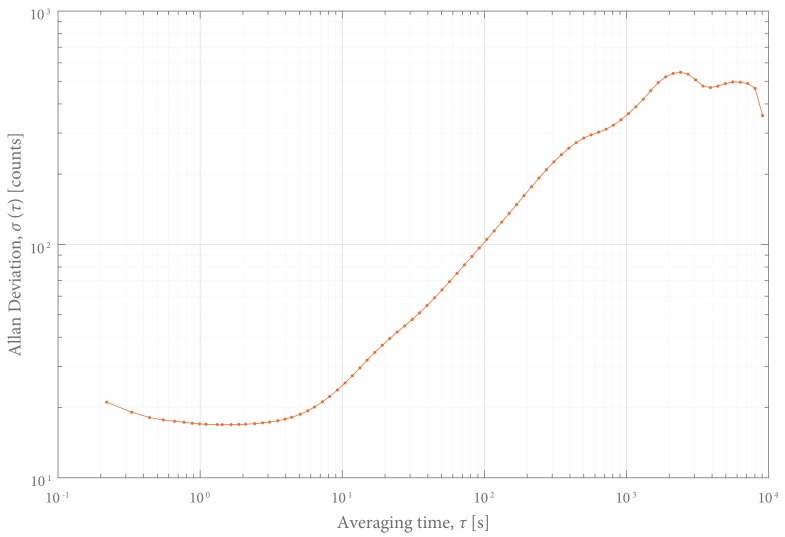
Allan deviation The brief decline at the onset of the curve indicates that the contribution of white noise is relatively small. The upward slope shows that the sensor response is dominated by drift. It can be concluded that the optimal averaging time is around 2 s.

The noise characteristics are presented in the Allan deviation graph in Figure 10. It shows that the contribution of white noise is relatively small and that the sensor response is dominated by drift.

### Total Error

The greatest sources of error are hysteresis (both ways, ±2.5%) and drift (±2.1%/log${}_{10}$(h)). When the output signal is corrected for the start-up effect and for the influence of temperature, the combined error is approximately ±5% of the full scale output. Depending on the application, the actual error might be higher due to influences that were not present in the tests used in this study.

## 5. Discussion

### 5.1. External Influences

A negative linear relationship was observed between a change in temperature and the output signal. This means that, with an temperature increase, the output signal suggests a larger distance between the LC-tank and the aluminum target. Thermal expansion of the rubber medium might be a cause for the observed temperature drift.

Most of the fluctuations of the sensor signal under a constant load could be explained by changes in temperature ($R^{2}$ > 0.84) and the signal was corrected for this influence. However, some residual fluctuations could be observed—other than noise or drift—with an unknown source. These fluctuations might be caused by higher order effects of temperature or by any other external influence, but this was not studied extensively.

### 5.2. Effective Resolution

The LHR_DATA consists of 28-bit numbers, but the resolution of the full output range is closer to 23–24 bit. This is due to 20–22 count differences between possible sample values. This quantization fits the documentation of Oberhauser [18], which predicts an LDC resolution of 3 Hz (corresponding to ∼20 counts in our reading) when an external oscillator of 40 MHz is used. As the sensitivity is specified using LHR_DATA, the effective resolution is 20–22 times the reciprocal of the sensitivity.

The sensors were calibrated over a range of 10 N, which generated an output signal covering approximately 1% of the 28-bit LDC range. This means that over the relevant force used, the sensor signal spans a 16-bit range. In practice, the sensor is able to detect changes in load in the range of tens of mN.

### 5.3. Drift

Although drift is a well-known phenomenon, different methods exist for its quantification. Because of its apparent randomness, it can be difficult to give a clear figure for drift. It is presented as a percentage after a specific time [3,4] or as a percentage per logarithmic hour [16,19,20].

Reports on force sensors with similar designs do not present a characterization of drift [8] and did not allow for any meaningful comparison, so we have compared the observed drift in our sensor to that of commonly used force sensors. We chose Parmar et al.’s [3] definitions and methods, who evaluated several force sensitive resistors (FSRs) and applied 72.7 mmHg to their sensors. After 8 h, the signal had drifted with 10–25% compared to the value after an initial settling time of 26 s. Using the same protocol Khodasevych et al. [4] found 0.4–24% drift after 8 h for more FSRs. In our static load test, a weight of 500 g was placed on the sensor with a 10 mm diameter, equal to 74.6 mmHg. After 8 h, our sensors showed 5.0 ± 1.4% drift.

The chosen *settling time* has a large influence on the figure for drift. Every sensor has some transient behavior before reaching its steady state, but the exact end of the transient behavior is hard to find and might be after several hours. The choice for a settling time of 26 s [3] or 30 s (our study) is arbitrary and creates a risk of bias. In most cases, drift is not purely linear but rather logarithmic, so the choice for a longer settling time generates a lower value for drift. Additionally, a longer settling time creates a higher reference value ($P_{i}$ in Parmar et al. [3]), which in turn also lowers the value for drift.

Figure 7 shows an apparent time response (whether it be called response time or drift) after every change in load in the incremental load test. While other sensors might have a clear distinction between response time and drift, our sensor does not: these characteristics rather seem to overlap. Due to drift of the sensor signal, it is impossible to determine a stable “end” value without choosing an arbitrary time span. It is therefore also not possible to determine how long it takes to reach this value. With the drift present, any calculation of response time would be influenced by the choice for an arbitrary time span and is therefore omitted in this paper. Our choice for a settling time of 30 s is arbitrary and of pragmatic nature.

Any quantification of drift should be practical, and presenting it as a percentage per logarithmic time gives a better idea about the error that has to be taken into account when studying the sensor data. According to the datasheets of various FSRs [19,20], their drift is <5%/log${}_{10}$(h). Using the same calculation method, our sensor shows a logarithmic drift of (2.1 ± 0.7) %/log${}_{10}$(h).

### 5.4. Reflection

For the specific application of long-term force measurements in the treatment of clubfoot, the performance of the developed force sensor fits most requirements. The sensor is thin enough to be placed underneath the cast without causing skin problems, and the AU is neither bulky nor heavy. The observed drift is better than for most FSRs [3,19,20] but slightly higher than required. In our application, the force is expected to slowly decrease to a certain minimum level. In those measurements, the error caused by drift might be limited since the observed drift appears to be load-dependent.

The influence of temperature cannot be corrected perfectly and some residual error can be expected. However, the temperature underneath a plaster cast is not expected to fluctuate much and by using an external thermometer most of the temperature influence can be corrected in post-processing. Hysteresis cannot be corrected for since the actual load on the sensor is unknown and therefore the loading history cannot be compensated for.

All sensors were assembled by hand which surely has introduced some variations in the amount of glue and subsequent distance between the LC-tank and the aluminum target. Any excessive glue might have created unforeseen connections between the rubber medium and the LC-tank, which is likely to have created variations in drift and hysteresis. Still the differences between the sensors tested were fairly small. A more controlled production method will decrease these differences and can be expected to improve the reproducability of the performance of the sensors.

This sensor is very suitable for other biomedical applications where gradual changes of force should be measured in a small volume. Applications that come to mind are prosthetic socket fitting and prevention of pressure ulcers. For applications requiring a higher bandwidth, a sacrifice in power consumption should be made in the settings of the LDC1614. For applications requiring other force ranges, the stiffness of the medium may be changed.

### 5.5. Limitations

We did not have the means to perform the tests in an environment with a controlled temperature, barometric pressure, and humidity. Instead we used the fluctuations in temperature to determine its influence and correct the signal for it. This may have introduced a confirmation bias. Additionally, the temperature influence was determined with a load of 500 g, and we did not test if this influence was load-dependent.

During the incremental load test, the maximum load (1 kg) was usually applied longer than the other loads. Due to the time drift, this might have lead to an overestimation of the calculated hysteresis.

### 5.6. Sensor in Practice

Preliminary results of measurements in the treatment of habitual toe walkers, Dupuytren fingers [21], and clubfeet [22] show excellent functioning for long-term measurement. Encountered problems in these applications were of a practical nature and were resolved with improved design: delamination of the aluminum target at the removal of the sensor was resolved by adding a post-it on top, battery failure was resolved by enhanced feedback with two LEDs on the AU and problems with identifying the start of important events in the logging was resolved by adding an input button on the AU.

## 6. Conclusions

A practical small-sized force sensor has been developed for long-term precision measurements in a portable setting. The used approach of inductive sensing combined with a compressible medium shows good applicability for the proposed force measurements between the skin and a cast, and great potential for many other applications.

## Figures and Tables

**Table:** 

	
(**a**)	(**b**)

**Table 1:** Sensor requirements. A small Lipo battery (165 mAh) should be able to power the system for at least one week (168 h), hence the set requirement for the maximum power consumption.

Requirements		Value	Unit
Dimensions	max	3 × 10 (ø)	mm
Dynamic range	min	0–10	N
Resolution	max	0.01	N
Time drift	max	1	%/day
Bandwidth	min	0.1	Hz
Current consumption	max	1.0	mA@3.7 V

**Table 2:** **Sensor performance**. All characteristics are presented as mean ± standard deviation.

	Static Load Test	Incremental Load Test
	(*n* = 9)	(*n* = 13)
Sensitivity ($10^{5}$ counts/N)	1.7 ± 0.8	1.3 ± 0.6
Non-linearity (%)		12 ± 10
Accuracy (%)		3.5 ± 3.0
Hysteresis (%)	5.8 ± 1.4	4.9 ± 2.1
Noise ($10^{-3}$ N)	1.2 ± 0.4	
Effective Resolution ($10^{-3}$ N)	0.15 ± 0.06	
Time drift (%/log${}_{10}$(h))	2.1 ± 0.7	
Time drift after 8 h (%)	5.0 ± 1.4	
Temperature drift (N/${}^{\circ }$C)	−0.088 ± 0.030	

## Abbreviations

The following abbreviations are used in this manuscript:

OEM	Original Equipment Manufacturer
FSR	Force Sensitive Resistor
LDC	Inductance-to-Digital Converter
LC-tank	Inductor Capacitor tank
PCB	Printed Circuit Board
AU	Acquisition Unit (see Figure 2)
SLU	Sensing and Logging Unit (see Figure 2)
